# Neutrophil extracellular traps induce endothelial damage and exacerbate vasospasm in traumatic brain injury

**DOI:** 10.7150/thno.115746

**Published:** 2025-08-16

**Authors:** Jinchao Wang, Lei Li, Jianye Xu, Dilmurat Gheyret, Kaiji Li, Xu Zhang, Haoran Jia, Ye Tian, Xiao Liu, Shenghui Li, Guili Yang, Yalong Gao, Ruilong Peng, Huajie Liu, Bin Liu, Jianfeng Zhuang, Cong Wang, Xin Chen, Yafan Liu, Bo Chen, Chuan Huang, Yuhan Li, Xin Chen, Jianning Zhang, Shu Zhang

**Affiliations:** 1 Department of Neurosurgery, Tianjin Medical University General Hospital, Tianjin, China.; 2 National Key Laboratory of Post-Trauma Neuro-Repair and Regeneration in Central Nervous System, Tianjin Key Laboratory of Injuries, Variations, and Regeneration of Nervous System, Tianjin Neurological Institute, Ministry of Education, Tianjin, China.; 3 Department of Neurosurgery, Qilu Hospital, Cheeloo College of Medicine and Institute of Brain and Brain-Inspired Science, Shandong University, Jinan, China.; 4 Shandong Key Laboratory of Brain Health and Function Remodeling, Jinan, China.; 5 Spine Center, Sanbo Brain Hospital, Capital Medical University, Beijing, China; 6 Medical College of Nankai University, Tianjin, China.; 7 Department of Neurosurgery, Tianjin Huanhu Hospital, Tianjin, China.; 8 Department of Neurosurgery, Shandong Provincial Hospital Affiliated to Shandong First Medical University, Shandong First Medical University, Jinan, China; 9 Department of Critical Care Medicine, Shandong Provincial Hospital Affiliated to Shandong First Medical University, Shandong First Medical University, Jinan, China; 10 Department of Physical Medicine and Rehabilitation, Tianjin Medical University General Hospital, 154 Anshan Rd, District Heping, Tianjin, China.; 11 State Key Laboratory of Experimental Hematology, Tianjin, China

**Keywords:** Traumatic brain injury, Neutrophil extracellular traps, Vascular spasm, TREM1, Endothelial dysfunction

## Abstract

Cerebral vasospasm (CVS) critically exacerbates secondary brain injury following traumatic brain injury (TBI). Understanding the underlying mechanisms is essential for developing targeted interventions.

**Methods:** We developed a comprehensive murine multimodal imaging platform to evaluate CVS cerebral perfusion, and blood-brain barrier (BBB) integrity, integrating *in vivo* multiphoton microscopy, magnetic resonance angiography, carotid Doppler ultrasound, and laser speckle contrast imaging with molecular assays and functional assessments. Additionally, we comprehensively analyze single-cell RNA (TBI vs Sham) and bulk-RNA data (NETs-treated vs Control), delineating NETs-driven endothelial injury signatures. Finally, we explored the roles of PAD4^-/-^, TLR4 inhibition and TREM1 blockade in blocking NETs-induced endothelial injury and CVS, validating key therapeutic targets.

**Results:** Our findings reveal that neutrophil extracellular traps (NETs) stimulate endothelial cells, promoting intracellular accumulation of TREM1, which forms a stable complex with NF-κB. This complex synergistically amplifies TLR4-mediated inflammatory responses, constituting a novel mechanism by which NETs aggravate endothelial injury and vasospasm after TBI. Preclinical interventions aimed at inhibiting NET formation or blocking TREM1 signaling significantly reduced neuroinflammation, cerebral edema, and CVS.

**Conclusions:** These findings identify TREM1 as a promising therapeutic target and illuminate a NET-driven crosstalk between vascular dysfunction and inflammatory cascades in the context of TBI, offering novel translational insights for mitigating secondary brain injury.

## Introduction

Traumatic Brain Injury (TBI) remains a leading cause of morbidity and mortality worldwide, particularly among individuals under 50 years of age, imposing a substantial burden on public health 1-3. The management of TBI presents unique challenges due to its heterogeneous nature, often involving complex pathological mechanisms. Following TBI, there is a defined temporal and spatial progression of injury, encompassing both primary and secondary brain damage 4. While primary injury occurs at the moment of impact and is largely irreversible, secondary injury develops over hours to days, presenting a critical window for therapeutic intervention. Disruption of the cerebral vascular system is a key driver of secondary brain injury, manifesting as cerebral vasospasm (CVS), blood-brain barrier (BBB) breakdown, and other vascular pathologies 5, 6. Despite its clinical significance, precise underlying pathological mechanisms remain incompletely elucidated.

Neutrophils, the most abundant immune cells in the humans, mount a rapid response following TBI and exert a complex dual role in the ensuing pathology 7. While they are indispensable for the acute immune response, forming specialized extracellular structures that entrap pathogens such as bacteria and viruses to mitigate infection 8 - excessive neutrophil activation and persistent inflammatory responses can contribute substantially to secondary neurological damage 9, 10. A key effector mechanism of neutrophil-mediated immune responses involves the release of neutrophil extracellular traps (NETs) web-like structures composed of extracellular double-stranded DNA, histones, myeloperoxidase, neutrophil elastase, thrombin, and other antimicrobial components 11, 12. Increasing evidence implicates dysregulation of NETs formation and excessive NETs release in the amplification of inflammatory responses 13, the pathogenesis of autoimmune disorders 14, and immunothrombosis^15^. In the central nervous system (CNS), multiple studies have shown the detrimental effects of NETs on neurons and glial cells, leading to adverse outcomes in various neurological disorders 16, 17. Our group has previously shown that inhibition of NETs formation in TBI models attenuates neuroinflammation, neuronal apoptosis, and neurological deficits 18. However, the specific impact of NETs on endothelial cell function and the underlying mechanisms remains to be elucidated.

Vasospasm is characterized by sustained segmental contraction of cerebral blood vessels in response to injury or external stimuli, leading to narrowing of the vascular lumen, reduced cerebral blood flow, and consequently, impaired neurological recovery 19, 20. As a form of secondary brain injury, CVS is closely associated with poor patient outcomes; however, its underlying mechanisms remain elusive. Current evidence suggests that TBI may directly compromise cerebral vascular endothelial integrity, leading to decreased production of vasodilatory factors (e.g., nitric oxide (NO) and prostaglandins), alongside increased release of potent vasoconstrictors (e.g., endothelin-1 (ET-1)), thereby inducing vasospasm 21. Additionally, inflammatory mediators released during neuroinflammatory cascades have been shown to stimulate smooth muscle contraction, triggering vasospasm 22, 23. Given their established roles in tissue injury and amplification of neuroinflammation, NETs are increasingly recognized as a potential novel driver of endothelial dysfunction and vasospasm following TBI.

In this study, we established a comprehensive murine assessment system for CVS and employed it to investigate alterations in cerebral endothelial function and the occurrence of CVS in PAD4^-/-^ mice following TBI. Leveraging this system, we further sought to elucidate the mechanisms by which NETs contribute to endothelial injury and CVS, with a focus on identifying key molecular mediators involved in these processes. Additionally, it is crucial to identify the mechanisms and key molecules involved in the role of NETs in CVS and endothelial injury. By delineating the interplay between neuroinflammation and endothelial dysfunction, this work aims to advance our understanding of secondary brain injury after TBI and to inform the development of targeted strategies to improve patient outcomes.

## Results

### CVS and BBB changes following TBI: A multimodal imaging study

**Figure 1:**
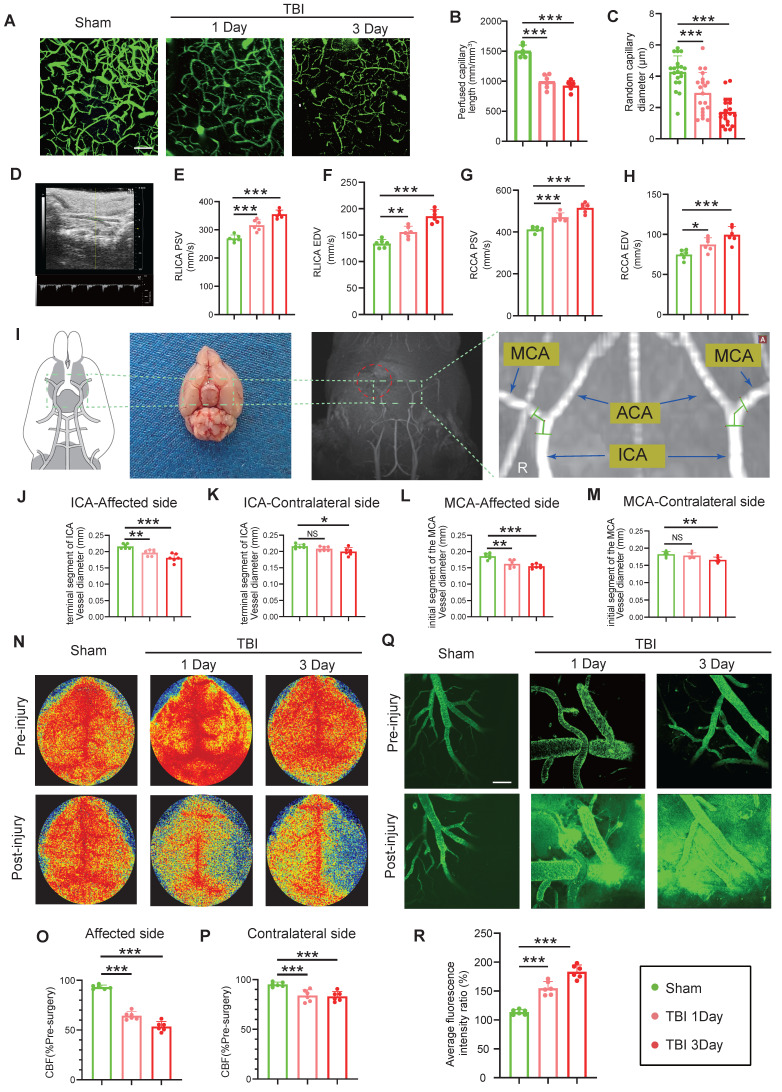
** Multimodal imaging assessment of CVS and BBB alterations following TBI. (A to C)** *In vivo* multiphoton microscopy images showing capillary perfusion in the contralateral hemisphere of the injured brain (n = 6/group) and diameters of 20 randomly selected capillaries per group (n = 6 mice/group), using FITC-dextran (MW = 2000 kDa) for capillary perfusion imaging. Bar = 100 µm. **(D)** Representative images of blood flow velocity measurements in the mouse carotid artery via ultrasound. **(E to H)** Quantitative Doppler ultrasound analysis of blood flow velocity, including PSV and EDV in the affected RCCA and RICA (n = 6/group). **(I)** MRI schematic showing the middle cerebral artery's starting segment and the internal carotid artery's terminal segment in mice, red highlights indicate injury sites in TBI models. **(J to M)** MRI-based measurements of intracranial vessel diameters in the affected ICA terminal segment, contralateral ICA terminal segment, affected MCA starting segment, and contralateral MCA starting segment (n = 6/group). **(N to P)** Representative images of CBF obtained through laser speckle measurements at 1 and 3 days post-TBI, along with quantitative analysis of traumatic injury and contralateral continuous CBF changes (n = 6/group). **(Q)** *In vivo* representative multiphoton microscopy images showing FITC-dextran (MW = 40 kDa, green) leakage in cortical vessels at Day-1 and Day-3post-TBI and in sham mice (n = 6/group). Bar = 100 µm. **(R)** Quantification of fluorescence intensity changes before and after TBI and sham treatment in the same vessels. Data are presented as mean ± standard deviation (S.D.) and analyzed by one-way or repeated measures analysis of variance (ANOVA) with Bonferroni's multiple comparison test as appropriate. *p < 0.05, **p < 0.01, ***p < 0.001.

*In vivo* multiphoton microscopy revealed the presence of CVS in vessels surrounding the cortical lesion site on Day-3 post-TBI, characterized by bead-like morphological changes and a reduction in vessel diameter (Figure S1A-B). Concurrently, *in vivo* multiphoton microscopy revealed a significant reduction in cortical microvascular perfusion post-TBI, with perfusion at Day-3 markedly lower than at Day-1, compared to the Sham group. This trend was consistent with the observed reductions in vessel diameter (Figure 1A-C). Doppler ultrasound analysis of carotid blood flow velocity showed significantly increased peak systolic velocity (PSV) in the affected right common carotid artery (RCCA) and right internal carotid artery (RICA) at Day-1 and Day-3 post-TBI, with more pronounced increases at Day-3. End-diastolic velocity (EDV) was similarly increased in both arteries (Figure 1D-H).

Magnetic resonance imaging (MRI) analysis revealed a substantial reduction in the vascular diameter of the ipsilateral internal carotid artery (ICA) and middle cerebral artery (MCA) at Day-1 and -3 post-TBI, with more severe narrowing at Day-3. The contralateral ICA and MCA showed no significant change on Day-1 but exhibited mild narrowing by Day-3 (Figure 1I-M). Laser speckle imaging demonstrated significantly reduced bilateral cerebral blood flow (CBF) at both time points, with a more pronounced reduction on the injured site, and at Day-3 (Figure 1N-P). Additionally, *in vivo* multiphoton imaging confirmed significantly higher FITC-dextran extravasation in the peri-lesional cortical region at Day-1 and Day-3 post-TBI relative to sham, with leakage intensifying at Day-3 (Figure 1Q, R). Evans blue (EB) extravasation assay corroborated these findings, showing increased EB leakage in wild-type (WT) mice at Day-1 and Day-3 post-TBI, with greater leakage at Day-3 (Figure S1C, D). MRI-based assessments demonstrated increased brain edema over the same period (Figure S1E, F).

### NETs deficiency alleviates CVS following TBI

**Figure 2:**
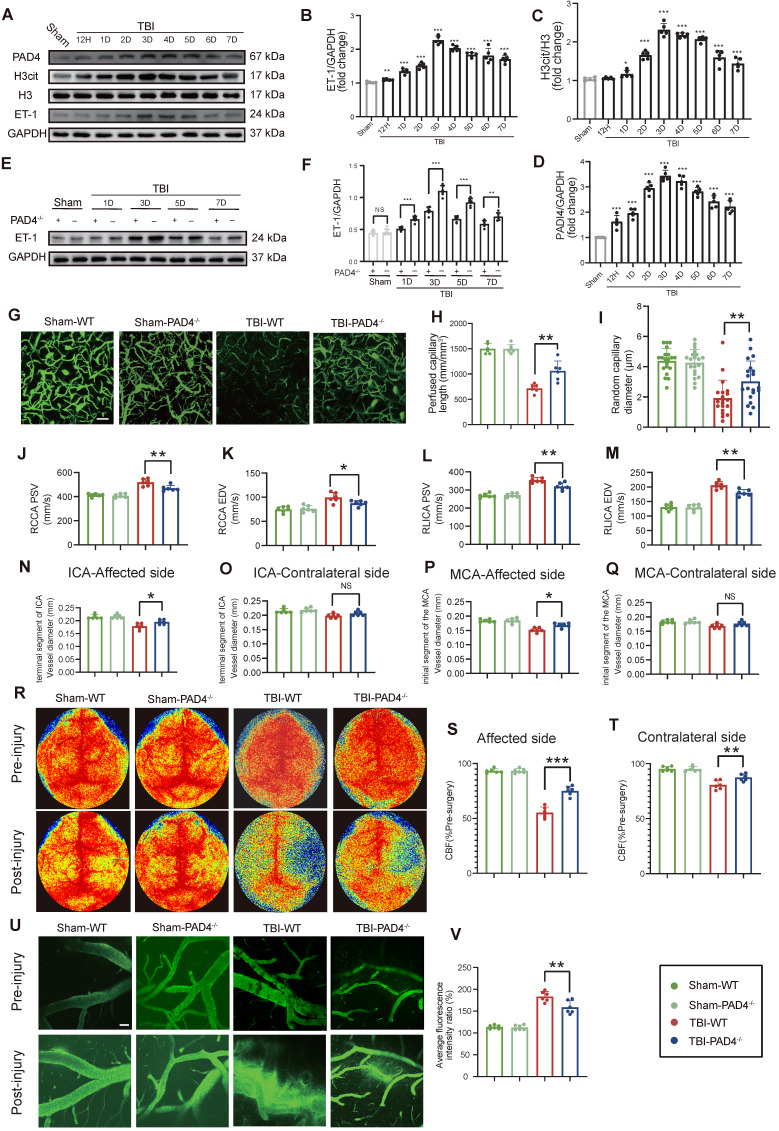
** NETs deficiency alleviates CVS following TBI. (A to D)** Western blot analysis and densitometric quantification of PAD4, H3cit, H3, and ET-1 expression in the injured cortex at various time points post-TBI compared to sham mice. **(E and F)** Western blot analysis and densitometric quantification of ET-1 expression in PAD4^-/-^ versus WT mice at multiple time points post-TBI, with sham-operated controls included for both genotypes. GAPDH served as the loading control. **(G to I)** Multiphoton microscopy images showing the quantification of perfused cortical capillaries (n = 6/group) and the diameters of randomly selected capillaries (n = 20/group) for capillary perfusion imaging in the hemisphere contralateral to the injury site. FITC-dextran (MW = 2000 kDa). Bar = 100 µm. **(J to M)** Quantitative ultrasound Doppler analysis of blood flow velocity, including PSV and EDV in the RCCA and RICA on the injured side. (n = 6/group). **(N to Q)** MRI analysis of intracranial vessel diameters in the affected ICA terminal segment, contralateral ICA terminal segment, affected MCA starting segment, and contralateral MCA starting segment (n = 6/group). **(R to T)** Representative laser speckle imaging of CBF at Day-3 post-TBI with quantitative analysis of continuous CBF changes in the injured and contralateral hemispheres (n = 6/group). **(U)** Representative multiphoton microscopy images showing leakage of FITC-dextran (MW = 40 kDa, green) in cortical vessels on day 3 post-TBI and in sham mice (n = 6/group). Bar = 100 µm. **(V)** Quantification of fluorescence intensity changes before and after TBI and sham treatment in the same vessels. (n = 6/group). Data are presented as mean ± S.D. and analyzed by one-way or repeated measures ANOVA with Bonferroni's multiple comparison test as appropriate. *p < 0.05, **p < 0.01, ***p < 0.001.

Temporal analysis revealed that NETs formation peaked at Day-3 post-TBI and exhibited a strong positive correlation with ET-1 release (r = 0.908; Figure 2A-D). Given that PAD4 is essential for NETs formation through by converting arginine to citrulline genetic ablation of PAD4 served to effectively suppress NET production 24, 25. Compared to WT TBI mice, PAD4^-/-^ TBI mice displayed significantly reduced ET-1 expression (Figure 2E, F). Correspondingly, cortical microvascular perfusion parameters, such as blood flow volume and capillary diameter, were markedly improved in PAD4^-/-^ TBI mice compared to WT TBI mice (Figure 2G-I). Doppler ultrasound analysis further confirmed this finding, demonstrating better preservation of both PSV and EDV, and improved carotid hemodynamics in PAD4^-/-^ TBI mice, demonstrating better preservation in major cerebral arteries (Figure 2J-M). MRI analysis revealed that PAD4 deficiency significantly mitigated the TBI-induced reduction in vascular diameters of the ipsilateral ICA and MCA, while vessel diameters on the contralateral side remained unchanged across groups (Fig. 2N-Q). Laser speckle imaging revealed that although TBI induced significant bilateral reductions in CBF, these decreases were significantly less pronounced in PAD4^-/-^ mice (Figure 2R-T). Importantly, *in vivo* multiphoton microscopy demonstrated markedly reduced fluorescence intensity from FITC-dextran leakage in peri-lesional cortical regions of PAD4^-/-^ TBI mice compared to WT TBI mice, indicating preserved vascular permeability (Figure 2U, V). EB assay also demonstrated a significant reduction in EB leakage in the PAD4^-/-^ mice at Day-3 post-TBI (Figure S2A), while MRI-based assessments similarly demonstrated attenuated brain edema (Figure S2B).

To evaluate long-term functional outcomes following TBI, we assessed spatial cognition using the Morris water maze (MWM), comprehensive neurological function via the modified neurological severity score (mNSS), and motor coordination with the Rotarod test. During MWM acquisition phase (Day 15-20), escape latency progressively decreased in all groups but remained significantly prolonged in TBI mice compared to Sham control. Notably, PAD4^-/-^ TBI mice showed markedly shorter escape latencies than WT TBI mice especially on Day-19 and -20 (Figure S1G, H). On Day-21, PAD4^-/-^ TBI mice demonstrated superior spatial memory, evidenced by reduced escape latencies, increased time spent in the target quadrant, and a higher number of platform crossings compared to WT TBI mice (Figure S1I, J). Furthermore, at Day-7 post-TBI, PAD4^-/-^ mice showed improved overall neurological function as indicated by lower mNSS scores, along with enhanced motor performance on the Rotarod compared to WT TBI counterparts (Figure S1K, L).

### NETs stimulate TREM1 expression and its interaction with the TLR/NF-κB pathway in endothelial cells

**Figure 3:**
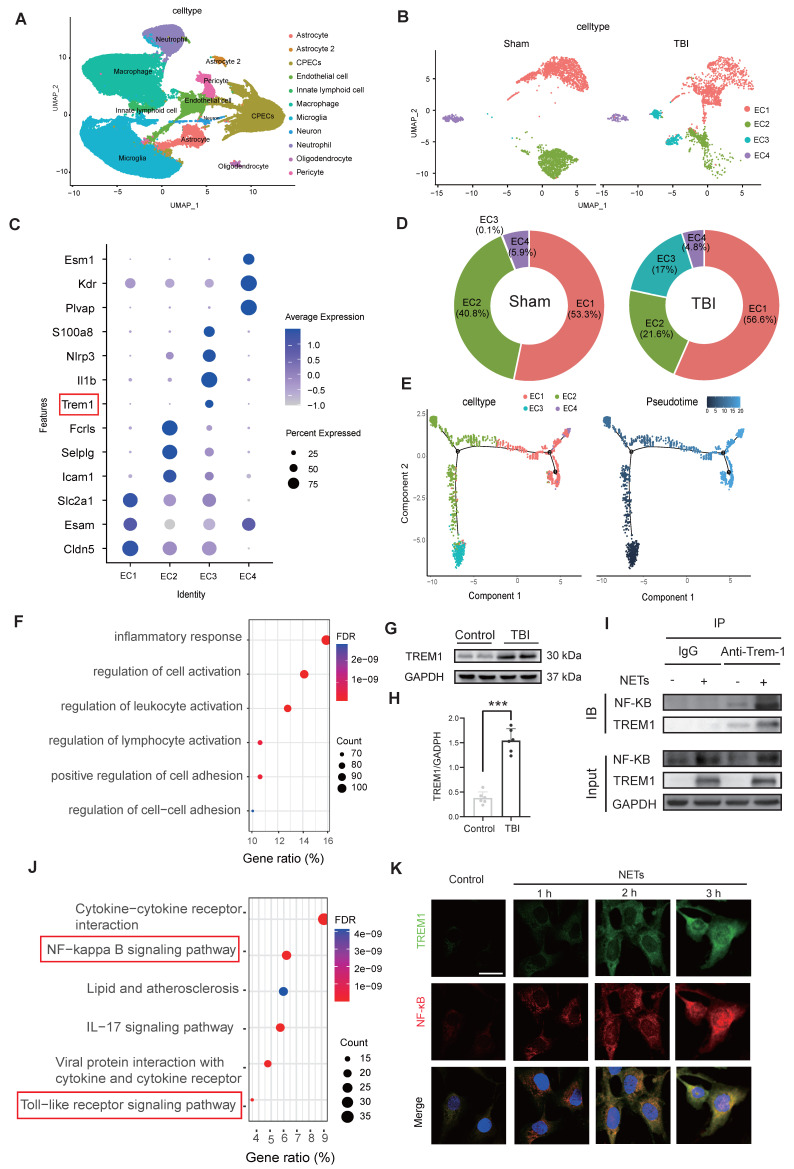
** NETs stimulate TREM1 expression and its interaction with the TLR/NF-κB pathway in endothelial cells. (A)** UMAP visualization of the expression differences in 124,595 single cells from injured and control mice, annotated by cell type based on established lineage markers. **(B)** Endothelial cells were extracted from the total cell population, followed by re-clustering and UMAP visualization, and separated into Sham and TBI groups. **(C)** Dot plot showing the expression of selected marker genes across different endothelial cell subpopulations. **(D)** Proportions of each endothelial cell population within different experimental groups. **(E)** Visualization of the results from pseudotime analysis of endothelial cells. **(F)** Visualization of the enrichment analysis of differentially expressed genes in the third endothelial cell cluster. **(G and H)** Western blot analysis and densitometric quantification were performed to evaluate the expression levels of TREM1 and GAPDH in patient samples. Data analyzed by unpaired *t*-test. (n = 6/group). **(I)** Co-IP results examining the physical association between NF-κB p65 and TREM1 in endothelial cells after NETs stimulation. **(J)** RNA-Seq analysis of injured cortical tissue from TBI mice, with functional enrichment of genes most strongly associated with TREM1 expression. **(K)** Representative images of double immunofluorescence staining for NF-κB (red) and TREM1 (green) in endothelial cells following different treatments. Nuclei were stained with DAPI (blue). (n = 6/group). Scale bar = 20 µm. Data are presented as mean ± S.D. and analyzed by one-way ANOVA with Bonferroni's multiple comparison test. *p < 0.05, **p < 0.01, ***p < 0.001.

Single-cell transcriptomic profiling of 124,595 cells from murine sham and TBI brain tissues identified 11 major cell clusters (Figure 3A). Endothelial cells were distinguished by the expression of canonical markers, including *Cldn5*, *Nos3*, and *Pecam1* (Figure S1M-O). Subsequent subclustering of 4,658 endothelial cells revealed four distinct subpopulations (Figure 3B). Analysis of signature gene expression patterns within these clusters allowed functional inference: EC1 represents typical mature brain microvascular endothelial cells that maintain the integrity of the blood-brain barrier; EC2 is involved in endothelial cell-cell adhesion and transmembrane transport; EC3 consists of endothelial cells activated under injury or inflammatory conditions; EC4 contains endothelial cells with characteristics of angiogenesis, fenestration, or specialized functional compartments(Figure 3C). Notably, TBI resulted in a significant expansion of the EC3 population (Figure 3D). Pseudotime trajectory analysis revealed the dynamic cellular progression with EC3 representing the terminal differentiation state (Figure 3E).

Functional enrichment of differentially expressed genes in the EC3 cell cluster are associated with inflammatory responses and regulation of cell activation (Figure 3F). Western blot analysis of patient samples confirmed a marked upregulation in TREM1 expression (Figure 3G, H). Co‑immunoprecipitation (Co‑IP) assays revealed that NETs stimulation promoted the interaction between TREM1 and NF-κB in endothelial cells (Figure 3I). Further, Pearson correlation analysis of the cortical RNA sequencing (RNA-Seq) from Day-3 post-TBI in mice, selecting the top 500 genes most strongly correlated with TREM1 (P-value < 0.01), revealed significant enrichment of the TLR/NF-κB signaling pathway (Figure 3J). Immunofluorescence confirmed that NETs stimulation induced a redistribution of TREM1 in endothelial cells shifting from its typical membrane localization to pronounced intracellular accumulation co-localized with F-actin structures (Figure S2C). Temporal and spatial expression patterns of TREM1 closely mirrored those of NF-κB following NETs stimulation (Figure 3K). To further validate these observations, subcellular fractionation of endothelial cells post-NET exposure indicated a slight increment of TREM1 expression in the membrane faction, while its expression was significantly elevated in both the cytoplasm and nucleus (Figure S2D-G).

### NETs inhibition modulates inflammation and tight junction protein expression in the TBI

**Figure 4:**
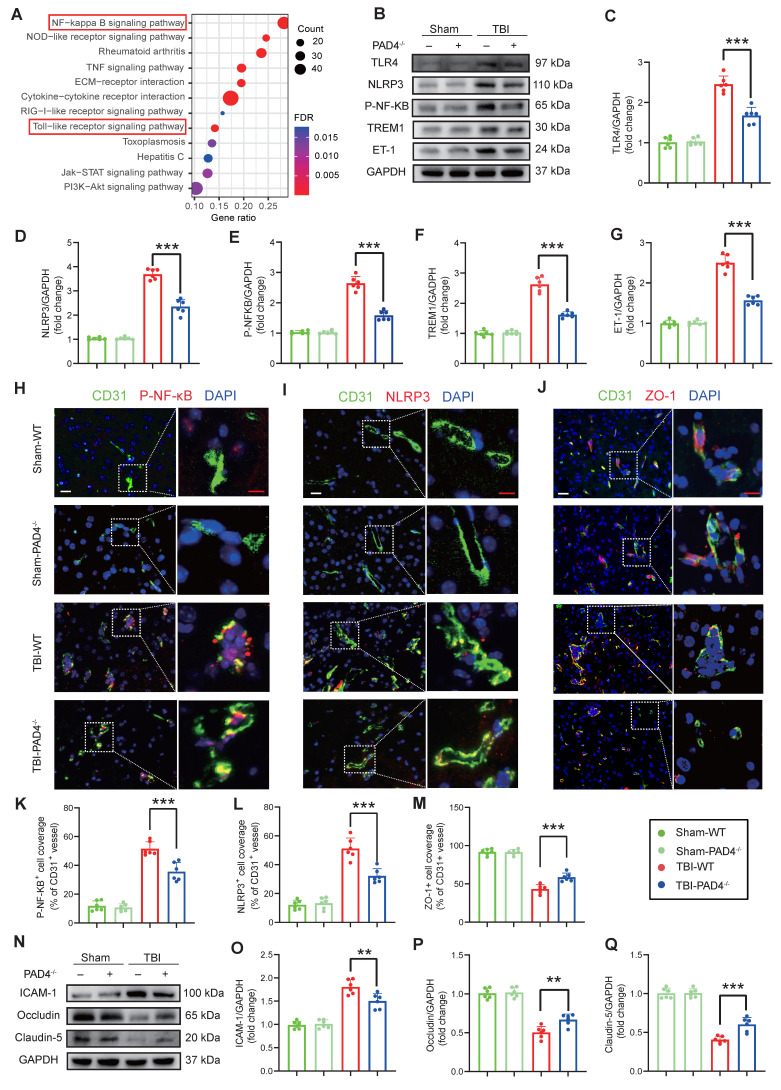
** NETs inhibition modulates inflammatory and tight junction protein expression in the TBI. (A)** KEGG pathway enrichment analysis of differentially expressed genes from RNA-Seq of NETs-treated and vehicle-treated HUVECs. **(B to G)** Western blot analysis and densitometric quantification were performed to measure the expression levels of TLR4, NLRP3, P-NF-κB, TREM1, ET-1 and GAPDH in the damaged cortex of TBI or sham group mice. (n = 6/group).** (H to M)** Representative images of double immunofluorescence staining for P-NF-κB (red), NLRP3 (red), or ZO-1 (red) and CD31 (green), along with quantitative analysis of mean fluorescence intensity, in the cortical injury sites of mice on day 3 post-TBI following different treatments. Nuclei were stained with DAPI (blue). White scale bar = 50 µm. Red scale bar = 20 µm. (n = 6/group). **(N to Q)** Western blot analysis and densitometric quantification were performed to measure the expression levels of ICAM-1, Occludin, Claudin-5, and GAPDH in the cortical injury sites of mice on day 3 post-TBI following different treatments. (n = 6/group). Data are presented as mean ± S.D. and analyzed by one-way ANOVA with Bonferroni's multiple comparison test. *p < 0.05, **p < 0.01, ***p < 0.001.

RNA-Seq revealed that NETs stimulation significantly activated the TLR/NF-κB pathway in endothelial cells compared to controls (FDR < 0.01; Fig. 4A), an effect that was abolished by treatment with the TLR4 inhibitor TAK242 (Figure S2H). Consistent with these in vitro findings, *in vivo* analyses at Day-3 post-TBI demonstrated that PAD4^-/-^ mice exhibited markedly reduced expression of TLR4, NLRP3, p-NF-κB, TREM1, and ET-1 compared to WT mice (Figure 4B-G). Immunofluorescence analysis of cortical tissue further confirmed these results, demonstrating decreased levels of p-NF-κB and NLRP3 in PAD4^-/-^ mice with an increase in the tight junction protein ZO-1 (Figure 4H-M). Western blot analysis showed concomitant improvements in blood-brain barrier integrity: decreased ICAM-1 with elevated Occludin and Claudin-5 in PAD4^-/-^ mice injured cortex (Figure 4N-Q).

### Inhibition of TREM1 and TLR4 ameliorates TBI endothelial dysfunction

**Figure 5:**
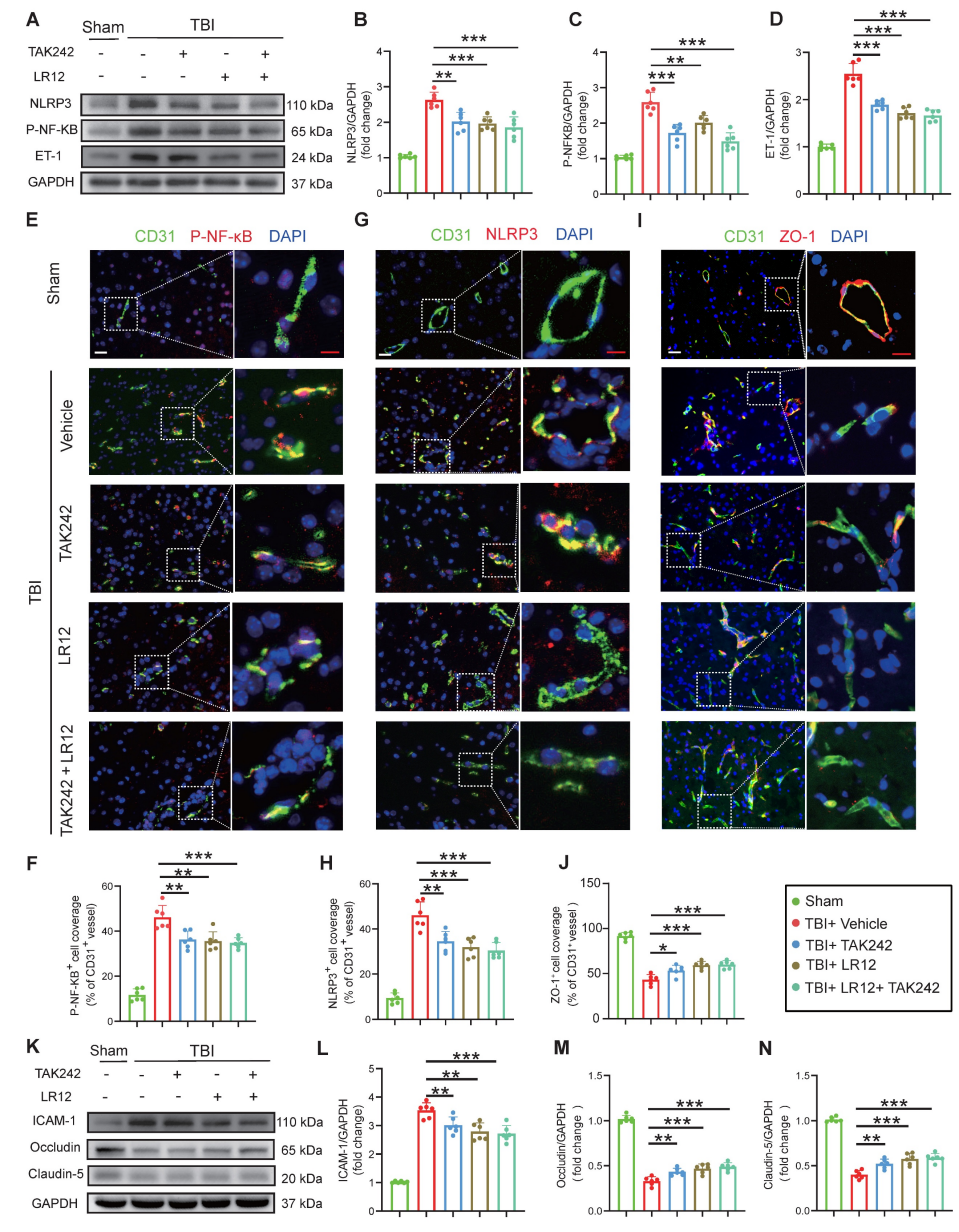
** Inhibition of TREM1 and TLR4 ameliorates TBI-induced endothelial dysfunction. (A to D)** Western blot analysis and densitometric quantification of NLRP3, P-NF-κB, ET-1, and GAPDH in the cortical injury sites of mice on Day-3 post-TBI following different treatments. (n = 6/group).** (E to J)** Representative images of double immunofluorescence staining for P-NF-κB (red), NLRP3 (red), or ZO-1 (red) and CD31 (green), along with quantitative analysis of mean fluorescence intensity, in the cortical injury sites of mice on Day-3 post-TBI following different treatments. Nuclei were stained with DAPI (blue). White scale bar = 50 µm. Red scale bar = 20 µm. (n = 6/group). **(K to N)** Western blot analysis and densitometric quantification were performed to measure the expression levels of ICAM-1, Occludin, Claudin-5, and GAPDH in the cortical injury sites of mice on Day-3 post-TBI following different treatments. (n = 6/group). Data is presented as mean ± S.D. and analyzed by one-way ANOVA with Bonferroni's multiple comparison test. *p < 0.05, **p < 0.01, ***p < 0.001.

*In vivo*, Western blot analysis and immunofluorescence quantification of cortical tissue samples from TBI mice treated with various drugs showed that treatment with LR12 (TREM1 inhibitor) and TAK242 monotherapy had similar effects in the effective inhibition of NLRP3, p-NF-κB, and ET-1, with the combination therapy producing an even more pronounced inhibitory effect (Figure 5A-H). Furthermore, immunofluorescence quantification and western blot analysis confirmed that these treatments also mitigated TBI-induced disruptions to endothelial tight junction proteins and adhesion molecules, including ZO-1, ICAM-1, Occludin, and Claudin-5. All treatment groups exhibited substantial protective effects against NET-induced endothelial injury, with LR12 monotherapy showing efficacy comparable to that of the combined TAK242/LR12 regimen (Figure 5I-N).

### *In vitro* validation of TLR4/TREM1 targeting in NETs-induced endothelial dysfunction

**Figure 6:**
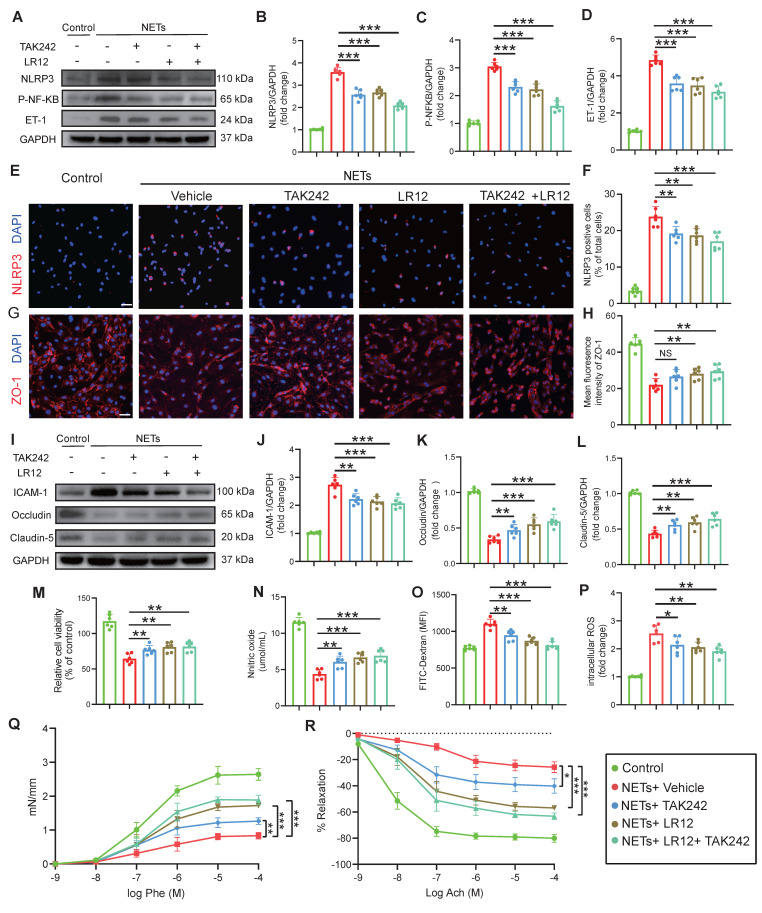
***In vitro* validation of TLR4/TREM1 targeting in NETs-induced endothelial dysfunction. (A to D)** Western blot analysis and densitometric quantification of NLRP3, P-NF-κB, ET-1, and GAPDH expression in endothelial cells under different treatment conditions. (n = 6/group). **(E to H)** Representative immunofluorescence images of NLRP3 (red) and ZO-1 (red)in endothelial cells under different conditions, with quantitative analysis of mean fluorescence intensity. Nuclei were stained with DAPI (blue). Scale bar = 50 µm. (n = 6/group). **(I to L)** Western blot analysis and densitometric quantification were performed to measure the expression levels of ICAM-1, Occludin, Claudin-5, and GAPDH in endothelial cells treated under different conditions. (n = 6/group). **(M)** *In vitro* HUVEC experiments were conducted to assess cell viability using the Cell Counting Kit-8 (CCK-8) assay (n = 6/group). **(N)** The culture media were also collected to quantify NO levels using a commercially available kit (n = 6/group).** (O)** Endothelial permeability was assessed by measuring the transendothelial leakage of FITC-dextran (70 kDa; n = 4/group). **(P)** Measurement of intracellular reactive oxygen species (ROS) levels in treated HUVECs (n = 6/group). **(Q and R)** Vascular ring tension assay generating concentration-response curves to phenylephrine (Phe) and acetylcholine (Ach) in carotid arteries from mice under different treatment conditions. Data are presented as mean ± S.D. and analyzed by one-way ANOVA or Two-way ANOVA with Bonferroni's multiple comparison test. *p < 0.05, **p < 0.01, ***p < 0.001.

Consistent with the *in vivo* findings (Fig. 5), in vitro studies on endothelial cells confirmed that both LR12 monotherapy and TAK242 monotherapy, as well as their combination, effectively suppressed NET-induced upregulation of NLRP3, phosphorylated NF-κB (p-NF-κB), and ET-1, as confirmed by Western blot analysis and quantitative immunofluorescence (Figure 6A-F). Importantly, both LR12 monotherapy and TAK242 monotherapy demonstrated comparable efficacy in restoring the loss of tight junction proteins (ZO-1, Occludin, Claudin-5) and reducing ICAM-1 expression following NETs stimulation, with the combination therapy yielded even greater protective effects (Figure 6G-L). These molecular findings were further supported by functional assays, which showed significant improvements in endothelial cell viability, NO production, attenuation of ROS release, and reduced endothelial permeability across all treatment groups (Figure 6M-P). Additionally, vascular ring tension assays revealed that NET-induced impairment of vascular function was markedly ameliorated in all treatment groups, with LR12 monotherapy and combination therapy demonstrating superior efficacy (Figure 6Q, R).

### Targeting of TREM1 and TLR4 improves CVS and functional outcomes after TBI *in vivo*

**Figure 7:**
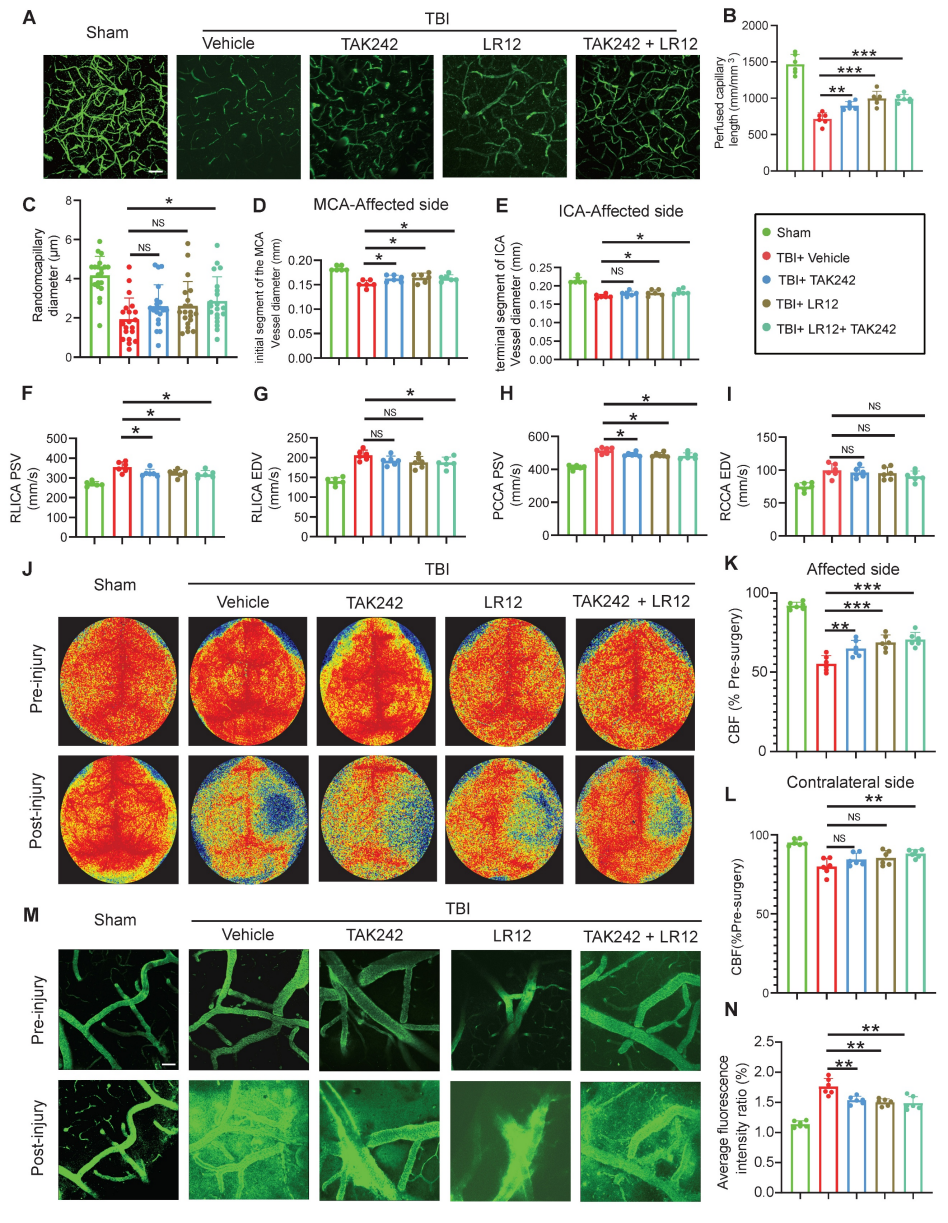
** Targeting TREM1 and TLR4 improves CVS and functional outcomes after TBI *in vivo*. (A to C)** Multiphoton microscopy images showing capillary perfusion in the contralateral hemisphere of the injured brain (n = 6/group), with quantification of diameters from 20 capillaries per group (n=6 mice/group), using FITC-dextran (MW = 2000 kDa). Bar = 100 µm. **(D, E)** MRI analysis of intracranial vessel diameters in the affected ICA terminal segment, affected MCA starting segment (n = 6/group). **(F to I)** Quantitative Doppler ultrasound analysis of blood flow velocity, including PSV and EDV in the affected RCCA and RICA (n = 6/group). **(J to L)** Representative laser speckle imaging of CBF at Day-3 post-TBI, with quantitative analysis of continuous CBF changes in both the injured and contralateral hemispheres (n = 6/group).** (M)** Representative multiphoton microscopy images showing leakage of FITC-dextran (MW = 40 kDa, green) in cortical vessels on Day-3 post-TBI and in sham mice (n = 6/group). Bar = 100 µm. **(N)** Quantification of fluorescence intensity changes in the same vessels before and after TBI or sham treatment. (n = 6/group). Data are presented as mean ± S.D. and analyzed by one-way ANOVA with Bonferroni's multiple comparison test. *p < 0.05, **p < 0.01, ***p < 0.001.

*In vivo* assessment on Day-3 post-TBI revealed that TAK242 monotherapy effectively alleviated the TBI-induced reduction in capillary perfusion compared to the Vehicle group, with even greater improvements observed in mice receiving either LR12 monotherapy or the LR12/TAK242 combination therapy (Figure 7A-C). Assessment of intracranial vessel diameters showed that all treatment groups (LR12, TAK242, combination) showed improved vessel diameter in the proximal segment of the ipsilateral MCA compared to the Vehicle group (Figure 7D). In contrast, in the distal segment of the ipsilateral ICA, TAK242 monotherapy failed to reverse vessel narrowing, whereas LR12 monotherapy and the combination therapy produced modest improvements compared to Vehicle (Figure 7E). Doppler ultrasound analysis demonstrated that all treatments) significantly reduced the elevated PSV in the ipsilateral CCA and ICA compared to the Vehicle group. However, only the combination therapy produced a slight improvement in the EDV of the ICA (Figure 7F-I). Laser speckle imaging further revealed significantly improved CBF on the injured ipsilateral side in the TAK242 monotherapy group, was even more pronounced improvements in the LR12 monotherapy and combination therapy groups. On the contralateral side, a significant improvement in CBF was observed only in the combination therapy group (Figure 7J-L). Assessment of vascular leakage via FITC-dextran extravasation showed that all treatment groups exhibited comparable and significant reductions in leakage compared to the Vehicle group (Figure 7M, N).

Extravasation assay performed on Day-3 post-TBI confirmed that all drug treatments significantly reduced EB leakage compared to Vehicle-treated TBI mice, with the combination therapy demonstrating the most pronounced reduction (Figure S2I). MRI-based assessment of brain edema revealed concordant results (Figure S2J). Behavioral testing with the MWM test on Day-21 revealed that only mice receiving the combination therapy spent significantly more time in the target quadrant and exhibited an increased number of platform crossings compared to Vehicle-treated TBI mice (Figure S2K-M). Assessment of comprehensive neurological function and motor performance on Day-7 post-TBI demonstrated significant functional improvement in all drug-treated groups compared to Vehicle controls, with LR12 monotherapy and the combination therapy yielded superior improvements compared to TAK242 monotherapy (Figure S2N, O).

## Discussion

In this study, we employed a novel multidimensional *in vivo* assessment strategy to comprehensively characterize vasospasm across multiple vascular compartments, including the cerebral surface vessels, cortical capillaries, MCA, ICA, and CCA *in vivo*. This approach enabled simultaneous evaluation of vessel diameters and flow velocities in large and medium-sized arteries, alongside capillary perfusion. Our findings revealed that upon NETs stimulation, TREM1, typically localized to the cell membrane, accumulates intracellularly and physically associates with NF-κB. This novel mechanism reveals that TREM1 mediates NETs-induced endothelial injury and vasospasm following TBI through a dedicated signaling cascade. This discovery not only elucidates the crucial role of the NETs-TREM1 axis in TBI-induced vasospasm but also highlights the dual function of TREM1 in bridging innate immune activation and cerebrovascular dysregulation. These findings offer new insights into the interaction between neuroinflammation and vascular dysfunction, provide a theoretical foundation for the development of targeted therapeutic strategies. Specifically, pharmacologic modulation of TREM1 to mitigate NET-associated vascular complications holds considerable translational promise in improving outcomes after TBI.

Following TBI, resident microglia become activated, initiating an inflammatory cascade that is further amplified by the infiltration of peripheral immune cells, such as neutrophils, culminating excessive neuroinflammation 26. Excessive neuroinflammation damages the BBB integrity, and BBB disruption in turn exacerbates neuroinflammation 27, creating a deleterious feedback loop. Despite numerous studies highlighting the detrimental effects of neuroinflammation following TBI, current clinical interventions remain limited, with anti-inflammatory treatments often proving ineffective 28. In fact, the CRASH trial revealed that high-dose glucocorticoid therapy could worsen the prognosis of TBI patients. Importantly, not all neuroinflammation is detrimental; certain aspects may support neural repair and recovery 29. Given these complexities, controlling excessive inflammation and mitigating factors that amplify the inflammatory response have become critical research directions. In our study, we focus on the detrimental effects of excessive inflammatory responses on endothelial cell injury as a key therapeutic target, aiming to alleviate secondary endothelial damage, brain edema, and vasospasm caused by TBI, ultimately improving patient outcomes. In contrast to the non-selective glucocorticoid treatment employed in the CRASH trial, our study achieves precise immune modulation by targeting the NETs-TREM1 axis. LR12 selectively inhibits excessive NETs signaling while preserving the fundamental defense functions of neutrophils, such as pathogen clearance, thereby mitigating the risk of systemic immune suppression.

During NETs formation, the release of a large amount of ROS directly induces oxidative damage to endothelial cells 30. Moreover, NETs are rich in proteases that can act directly on endothelial cells, promoting cellular activation and injury. For example, neutrophil elastase 31, myeloperoxidase 32, 33, and proteinase G 34 have been known to degrade cell junction proteins, activate endothelial cells, and disrupt vascular structure, further exacerbating endothelial damage. In this study, we identified a critical role for the TLR4/NF-κB signaling pathway in mediating NETs-induced endothelial injury, implicating TLR4 as a key sensor of NETs and initiating pro-damage signaling cascades. However, this does not preclude the direct contribution of NET-derived ROS and proteases as effector molecules in endothelial injury. Future investigations should explore the potential synergistic interactions among TLR4 signaling, specific NET-associated proteases, and ROS in orchestrating endothelial injury. Such studies could yield deeper mechanistic insights and inform the development of targeted therapeutic strategies to improve clinical outcomes in patients with TBI.

TREM1, widely expressed in monocytes and microglia, is well recognized for its role in amplifying oxidative burst and the secretion of pro-inflammatory cytokines 35. Previous studies on TREM1 have mainly focused on its role in immune cells in chronic diseases such as cancer and diabetes, where it acts as a pro-inflammatory receptor that enhances the inflammatory response 36, 37. In this study, we found that TREM1 expressionwas significantly upregulated in endothelial cells following TBI, as confirmed by both our mouse model and clinical patient samples. Our findings demonstrate that TREM1 physically interacts with NF-κB in response to NETs stimulation, forming a signaling node that markedly amplifies the inflammatory cascade downstream of TLR4 activation. This TREM1- NF-κB interaction represents a mechanism distinct from the parallel activation of TREM1 and TLR4 signaling observed in sepsis 38, suggesting a TBI-specific damage-associated molecular patterns co-recognition mechanism. Interestingly, NET stimulation led to aberrant intracellular accumulation of TREM1, deviating from its typical membrane localization, a process potentially linked to evasion of lysosomal degradation. This phenomenon resembles patterns observed in Alzheimer's disease models characterized by amyloid beta deposition 39. Given the central role of TREM1 signaling in NETs-induced responses observed in our study, we speculate that TREM1 accumulation affects the nuclear translocation of NF-κB, thereby modulating the expression of downstream genes. From a therapeutic perspective, our *in vivo* and *in vitro* data, consistently demonstrated that the TREM1 inhibitor LR12 often outperformed the TLR4 inhibitor TAK242 in alleviating NET-induced endothelial injury and vasospasm. Leveraging the advantages of LR12 in improving endothelial function, future strategies could involve the design of TREM1-specific nanobodies combined with BBB penetrating peptides for targeted central delivery. Additionally, co-administration of PAD4 inhibitors may synergistically enhance therapeutic efficacy by suppressing NETs generation at the source while blocking downstream signaling. Collectively, these findings provide a strong theoretical foundation for the development of dual-targeted therapeutic strategies aimed at mitigating secondary vascular and inflammatory damage following TBI.

Post-traumatic vasospasm is a critical pathological process in secondary brain injury, with incidence rates varying based on age, injury severity, and detection methods 40. In adults with severe TBI, reported incidence ranges from 27% to 63%, while in pediatric populations it is 3%-8.5%, but increases substantially to 21%-33.5% in severe cases 41. However, these data are likely underestimated, given that vascular monitoring is not routinely performed after TBI in many clinical settings, and transient, reversible episodes of CVS often go undetected. Transcranial Doppler ultrasound is the preferred non-invasive tool, whereas CT angiography and digital subtraction angiography are considered the gold standards for assessing the degree of vascular narrowing 40. Cerebral infarction caused by vasospasm significantly increases the risk of neurological deterioration, with more than a twofold increase in the risk of delayed cerebral ischemia and poor prognosis 40. Preclinical assessment of CVS in murine models lacks gold standards due to anatomical constraints. Prior studies relied on techniques including CT/MRI angiography, laser speckle contrast imaging, vasoactive substance detection, or vascular histology 22, 42, 43. To address this, we developed a multimodal platform combining: *in vivo* two-photon microscopy (vessel dynamics), vascular leakage quantification, small-animal MRI angiography, carotid Doppler ultrasound, and ET-1/NO detection. This comprehensive evaluation allowed us to thoroughly assess endothelial damage and vasospasm following TBI.

Vasospasm is a complex pathophysiological process that requires coordinated interactions among endothelial cells, pericytes, and vascular smooth muscle cells. While early mechanical injury from trauma can directly damage vascular smooth muscle, subsequent factors such as oxygenated hemoglobin and ET-1 further trigger inflammation and oxidative stress 44, 45. As early mechanical insult is often uncontrollable, research has increasingly focused on mitigating secondary brain injury driven by excessive inflammation. Merging evidence has highlighted a critical role for in this context. NETs have been shown to activate a pro-inflammatory phenotype in vascular smooth muscle cells, exacerbating vascular damage 46. Additionally, in TBI, NETs upregulate CD11b in pericytes, contributing to neuroinflammation and BBB dysfunction 47. Similarly, in stroke models, the release of NETs impairs endothelial function, affecting vascular remodeling during stroke recovery, including reduced angiogenesis and increased BBB damage 48. Our study corroborates these findings by demonstrating NET-mediated endothelial injury and vasospasm after TBI, while elucidating key mechanistic pathways involved. Moreover, we established a comprehensive framework for evaluating vascular pathology in TBI models, offering a robust platform for future mechanistic investigations and the development of targeted therapeutic strategies.

Several limitations of this study should be acknowledged. First, few preclinical studies have included both male and female animals 49. Given that TBI predominantly occurs in males, most current TBI studies have exclusively used male animals. However, reports of TBI in females are increasing, and there is growing interest in investigating sex differences in TBI pathophysiology 50. Second, all TREM1-blocking strategies employed in this study were systemic. Although we originally planned to generate an endothelial cell-specific TREM1 knockout model to directly validate the role of TREM1 within the vascular endothelium, this was not achieved due to experimental constraints. Additionally, the pharmacokinetics of LR12 and its BBB penetration efficiency require further optimization. Our study investigated the expression and potential function of TREM1 in endothelial cells, where it is also expressed in other cells such as microglia and neutrophils, underscoring its complex role in the immune system. As research on TREM1 is still in its early stages, additional studies are necessary to fully delineate its diverse functions and therapeutic potential. We plan to continue investigations in this area to address these important gaps.

## Methods

### TBI patients and samples

Between May 2022 and May 2024, we conducted a study involving human brain tissue collection under a research protocol that was thoroughly reviewed and approved by the Ethics Committee of Tianjin Medical University General Hospital. Written informed consent was obtained from all participants or their legal representatives. For the investigation of TBI, we selected a cohort of six male patients diagnosed with severe TBI, defined by a Glasgow Coma Scale (GCS) score of less than 9. All patients underwent necessary decompressive craniectomy to evacuate intracranial hematoma and/or alleviate life-threatening intracranial pressure. As controls, we included six male patients with no known history of neurological disorders. These patients underwent brain surgery for deep brain meningioma resection (four patients) or cerebrovascular malformation removal (two patients). Control brain tissues were collected from macroscopically and histologically confirmed normal brain regions adjacent to the surgical site during these procedures.

### Animals

In this study, male C57BL/6 mice, aged 8-16 weeks and weighing 22-30 g, were sourced from the Experimental Animal Research Center of the Academy of Military Medical Sciences, Beijing, China. Mice were acclimated to controlled conditions with a temperature range of 20-24°C, humidity maintained between 50% and 60%, and a 12-hour light/dark cycle. Food and water were provided *ad libitum*. PAD4^-/-^ mice on a C57BL/6J background were obtained from The Jackson Laboratory, with age-matched wild-type C57BL/6 mice used as controls. All procedures adhered to ethical standards and were approved by the Animal Care and Use Committee of Tianjin Medical University. Efforts were made to minimize animal usage and alleviate suffering.

### TBI model

In this study, a controlled cortical impact (CCI) model—one of the most widely utilized paradigms for experimental TBI—was employed to replicate clinical features of TBI in mice 51. Mice were anesthetized with 1-1.5% isoflurane in a mixture of 30% oxygen and 70% nitrous oxide. After securing each animal in a stereotaxic frame, a 3-mm craniotomy was performed on the right parietal cortex (2.5 mm posterior to the bregma and 2.5 mm lateral to the sagittal suture) with a high-speed microdrill. A 3-mm flat-impact tip was used to deliver a unilateral impact with a depth of 2.2 mm, a velocity of 5 m/s, and a dwell time of 200 ms to induce TBI. Post-injury, mice were randomly assigned to different experimental groups and received designated pharmacologic treatments. All surgical procedures were performed under strict aseptic conditions. Data collection and analyses were conducted by investigators blinded to group assignments.

### Cell lines, cell culture, and evaluation of cellular functions

Human umbilical vein endothelial cells (HUVECs; ATCC, Manassas, VA, USA) were cultured in Endothelial Growth Medium-2 (Allendale, NJ, USA) at 37°C in a humidified atmosphere with 5% CO₂, with medium changes every 2-3 days. NETs were administered at a concentration of 1 μg/mL, and after 24 hours of incubation, various assays were performed 52. Cell viability was evaluated using the CCK-8 assay (Cat. No. C0038; Beyotime, Jiangsu, China). Culture media were collected to measure NO levels (Cat. No. S0021; Beyotime) using commercial kits. The treated HUVECs were then harvested for intracellular reactive ROS measurement using the 2′,7′-dichlorofluorescin diacetate method (ROS assay kit, Cat. No. CA1410; Solarbio).

### Drug administration

To assess the effects of TREM1 inhibition, species-specific analogs of LR12 peptide were used: human LR12 (hLR12: LQEEDAGEYGCM) and murine LR12 (mLR12: LQEEDTGEYGCV). In mice, mLR12 (150 µg) was administered by intraperitoneal injection once daily for three days. For cellular inhibition, hLR12 was added to the culture at 25 µg/mL. The TLR4 inhibitor Resatorvid (TAK-242) was used to block TLR4 signaling. *In vivo*, TAK-242 was given intraperitoneally at 3 mg/kg for three days. *In vitro*, TAK-242 was added to cells at 5 µM, one hour before stimulation.

### Endothelial permeability assay

Endothelial permeability was measured via FITC-dextran (Cat. No. ECM644; Millipore) leakage across an endothelial monolayer. HUVECs were seeded on collagen-coated Transwell inserts (0.4 µm pore size) in a 24-well plate and cultured to confluence. After treatment under various conditions for 3 hours at 37°C, FITC-dextran was added to the upper chamber and incubated for 1 hour. The medium from the lower chambers was collected, and fluorescence intensity was quantified using a SpectraMax M5 plate reader (Molecular Devices) at 485 nm excitation and 535 nm emission. Fluorescence levels indicated changes in permeability.

### Magnetic resonance imaging

MRI was used to assess brain edema and intracranial vessel diameters in mice on Day-3 post-injury using a 9.4T animal scanner (Bruker Biospin, Germany) with a slice thickness of 0.5 mm. Brain edema was quantified by measuring hyperintense areas on T2-weighted images. Intracranial vessel diameters were assessed using the TOF-3D-FLASH sequence, with lumen diameter measured 0.2 mm distal to the internal carotid artery bifurcation. Image analysis was performed with Weasis software 53.

### Laser speckle contrast imaging

To evaluate changes in CBF before surgery and at Day-3 post-CCI, we used a laser speckle contrast imaging system (PeriCam PSI System; Perimed). Mice were deeply anesthetized and positioned in a stereotaxic frame, followed by a midline incision to expose the skull. CBF was continuously measured for 60 seconds at a height of 10 cm, with the region of interest defined as the same position of the brain on both the injury site and the contralateral side. CBF changes were quantified using PIMsoft software (version 1.4; Perimed). Data collection and analyses were conducted by investigators blinded to group assignments.

### Neutrophil isolation and *in vitro* induction of NETs formation

Peripheral blood was collected in sodium citrate anticoagulant tubes, and neutrophils were isolated using a neutrophil isolation kit (MACSxpress® Whole Blood Neutrophil Isolation Kit, human 130104434, Milteny). The neutrophil-enriched fraction was collected and treated with red blood cell lysis buffer to remove residual erythrocytes. Purified neutrophils were resuspended in RPMI-1640 medium, supplemented, and stimulated with 10 μg/mL LPS (Sigma-Aldrich, MO, USA) for 4 hours at 37°C under 5% CO₂. After incubation, the supernatants were centrifuged at 300 × g for 5 minutes to eliminate cells and debris. The supernatants were then collected and stored at -80°C for subsequent analysis.

### RNA-Seq and subsequent bioinformatics analyse

Total RNA was extracted and purified from treated HUVECs and the peri-lesional cortex using TRIzol reagent (15596018CN; Invitrogen) according to the manufacturer's instructions. A paired-end library was constructed according to the TruSeq® RNA Library Preparation Guide. Briefly, mRNA was purified using magnetic beads, fragmented, and reverse transcribed into double-stranded cDNA. Libraries were then generated through end repair, adaptor ligation, and PCR amplification. Library quality was assessed with Qubit fluorometer and Agilent Bioanalyzer, while the diluted library was clustered using cBot and sequenced on the Illumina HiSeq Xten (performed by Shanghai Biotechnology Corporation). To assess the biological function of individual genes, we applied Pearson correlation analysis and selected the top 500 most correlated genes with a P-value < 0.01. KEGG pathway enrichment analysis was then performed using the “clusterProfiler” package.

### Western blot analysis

Western blot analysis was performed to detect and quantify the relative expression of specific proteins. Briefly, brain tissue and cell samples were harvested, and total protein was extracted using RIPA buffer supplemented with protease and phosphatase inhibitors to prevent degradation. Cell membrane proteins, cytoplasmic proteins, and nuclear proteins were extracted separately using kits from Beyotime (P0033 and P0028). Protein samples were separated by SDS-PAGE and transferred onto PVDF membranes. The membranes were blocked with 5% non-fat milk for 2 hours, followed by overnight incubation at 4°C with primary antibodies targeting proteins such as ICAM-1 (Cat. No. ab171123; Abcam), Occludin (Cat. No. ab216327; Abcam), Claudin-5 (Cat. No. A25830; ABclonal), NLRP3 (Cat. No. 68102-1-Ig; Proteintech), GAPDH (Cat. No. ab8245; Abcam), PAD4 (Cat. No. ab96758; Abcam), H3Cit (Cat. No. ab5103; Abcam), Histone H3 (Cat. No. 9715; Cell Signaling Technology), NF-κB (Cat. No. ab16502; Abcam), Phospho-NF-κB (Cat. No. AF3392; Affinity) ET-1 (Cat. No. 12191-1-AP; Proteintech), TLR4 (Cat. No. A5258; ABclonal), TREM1 (Cat. No. 11791-1-AP; Proteintech). After washing, the membranes were incubated for 1 hour at room temperature with horseradish peroxidase-conjugated secondary antibodies. Protein bands were detected using enhanced chemiluminescence, and the signal intensity was quantified using ImageJ software.

### Immunofuorescence staining

Tissue samples were fixed via transcardial perfusion with 4% paraformaldehyde (PFA), post-fixed overnight, dehydrated in 20%/30% sucrose, embedded in OCT compound, and sectioned coronally (7 μm). For cellular analysis, cells on coverslips were fixed with 4% PFA (20 min). Sections/cells were permeabilized with 0.1% Triton X-100 and blocked (10% goat serum or 3% BSA), incubated overnight at 4°C with primary antibodies CD31 (Cat. No. Ab182981; Abcam), phalloidin (Cat. No. CA1620; Solarbio), NLRP3 (Cat. No. 68102-1-Ig; Proteintech), NF-κB (Cat. No. ab76302; Abcam), Phospho-NF-κB (Cat. No. AF3392; Affinity), ZO-1 (Cat. No. Ab281583; Abcam), followed by Alexa Fluor 488/594-conjugated secondary antibodies (1h, RT) and DAPI counterstaining. Apoptosis was assessed using a one-step TUNEL kit (C1089, Beyotime). Images were acquired via confocal or fluorescence microscopy and quantified with ImageJ software.

### Ultrasound detection of carotid artery diameter

Mice were subjected to carotid ultrasound (Vevo 2100, VisualSonics) on Day-3 post-TBI under isoflurane anesthesia (3.5% induction, 1.5% maintenance). After depilatory preparation, sagittal-plane imaging of bilateral carotid bifurcations was performed with ultrasound gel-enhanced probe alignment. Vessel diameters were measured: 1.5 mm upstream (common carotid) and 0.5 mm downstream (external/internal branches) of the bifurcation. Triplicate measurements at each site were averaged across systolic/diastolic phases (3 cardiac cycles).

### Vascular reactivity assay

Common carotid arteries from euthanized mice were incubated in RPMI medium with 10 μg NETs ± mLR12/TAK242 (3 hrs) and transferred to ice-cold physiological saline (130 mM NaCl, 14.9 mM NaHCO₃, 3.7 mM KCl, 1.2 mM MgSO₄, 2.5 mM CaCl₂, 1.2 mM KH₂PO₄, 5.5 mM glucose). 2-mm rings were mounted on a myograph (40 μm wires), perfused with carbogen (95% O₂/5% CO₂). After 20-min equilibration, maximal contraction was tested with 100 mM KCl and 1 μM phenylephrine (Phe). Cumulative Phe (1 nM-100 μM) and acetylcholine (Ach, 1 nM-100 μM) dose-response curves were generated post-washout, with precontraction to 80% Phe-induced maximum for Ach evaluation.

### *In vivo* multiphoton imaging

To visualize cortical blood vessels and evaluate permeability and cerebral perfusion, a cranial window was surgically created over the injury site and the left somatosensory cortex. FITC-dextran tracers (40 kDa and 2000 kDa; 0.1 mL, 2.5 mg/mL each) were intravenously administered to assess BBB permeability and cerebral perfusion. Imaging was performed using a FluoView FVMPE-RS multiphoton system with a 25× objective (500×500 μm field of view). Vascular permeability and microvascular perfusion were dynamically monitored at fixed intervals (every 180 seconds) over 30-minute sessions. To evaluate microvascular perfusion, Z-stack imaging was acquired with a 5 μm step size, spanning depths of 300 to 500 μm beneath the cortical surface. Image analysis was conducted using ImageJ software. Specifically, the total length of microvessels with diameters <6 μm was quantified and expressed as vessel length (mm) per cubic millimeter of cortical tissue.

### Co‑immunoprecipitation

Cell lysates prepared with IP buffer were incubated overnight at 4°C with anti-TREM1 antibody-conjugated magnetic beads (Pierce Classic IP/Co-IP Kit #88804; Thermo). After magnetic separation and three wash cycles, bound complexes were eluted using 1× SDS-PAGE buffer (95°C, 5 min) for subsequent immunoblotting and LC-MS/MS proteomic analysis (Q Exactive™ HF-X system).

### Single cell sequencing analysis

Single-cell RNA sequencing data were obtained from the GEO database (accession number GSE269748) (https://www.ncbi.nlm.nih.gov/geo)^54^. Barcodes, feature files, and count matrices for each sample were used to generate Scanpy objects. Genes expressed in fewer than three cells and cells with fewer than 200 genes were excluded. Additional quality control was performed based on the number of genes detected in the count matrix and the percentage of mitochondrial gene expression. High-variance genes were selected, and cell cycle phase information was calculated. Batch effects were corrected using the Harmony algorithm. Dimensionality reduction, clustering, and visualization were performed using the FindNeighbors, FindClusters, and UMAP/t-SNE functions, with a resolution of 0.5. Cell annotations were based on the CellMarker 2.0 database. Endothelial cell subpopulations were further re-clustered and analyzed. Pseudotime trajectories were inferred using Monocle 2, and pseudotime-dependent gene expression dynamics were characterized with scEpath^55^. To model gene expression changes in pseudotime, scEpath first divides pseudotime into 10 equally spaced bins. The expression of each gene in each bin is then estimated by the trimean of gene expression values across all cells located in that bin. Additionally, scEpath smooths the average expression of each gene using cubic regression splines. To identify significantly changed pseudotime-dependent genes, the standard deviation of the observed smoothed expressions was compared with that of permuted expressions generated by randomly permuting cell order (1000 permutations in our analysis). Genes with a standard deviation greater than 0.5 and a Bonferroni-corrected p-value below a significance threshold of α = 0.01 were considered to be pseudotime-dependent. Differentially expressed genes obtained from the single-cell analysis were subjected to functional enrichment analysis using the Metascape database (https://metascape.org).

### Statistical analysis

Data were expressed as the mean ± S.D. Variance homogeneity was evaluated using the Levene's test, and the normality of the quantitative data was assessed with the Kolmogorov-Smirnov test. When data were assumed to be normally distributed with homogeneous variances, pairwise comparisons were performed using Student's *t*-tests, while appropriate ANOVA models - including one-way ANOVA, repeated measures ANOVA, or two-way ANOVA - were employed for multi-group comparisons, followed by Tukey's honestly significant difference *post-hoc* test for multiple comparisons correction. Non-parametric comparisons between groups were conducted using the Kruskal-Wallis test. Correlations were assessed via Pearson's method. The statistical analyses were conducted using SPSS software (version 26.0, IBM Corporation). A p-value below 0.05 was recognized as statistically significant.

## Supplementary Material

Supplementary methods and figures.

## Abbreviations

CVS: cerebral vasospasm
TBI: traumatic brain injury
NETs: neutrophil extracellular traps
BBB: blood-brain barrier
NO: nitric oxide
ET-1: endothelin-1
ICA: internal carotid artery
MCA: middle cerebral artery
PSV: peak systolic velocity
RCCA: right common carotid artery
EDV: end-diastolic velocity
RICA: right internal carotid artery
ROS: reactive oxygen species
CCI: controlled cortical impact
ANOV: analysis of variance
