# Supplementary material for: Neutrophil extracellular traps induce endothelial damage and exacerbate vasospasm in traumatic brain injury
Source: Theranostics. 2025 Aug 16;15(17):9221–39. doi: 10.7150/thno.115746 (PMC12439479; doi:10.7150/thno.115746)
Supplement: Supplementary file 1 — Supplementary methods and figures. [file thnov15p9221s1.pdf]

Supplementary methods and materials

EB Dye Extravasation Assay

To evaluate BBB permeability, an EB dye extravasation assay was conducted. Mice were intravenously injected with 2% EB dye (4 mL/kg, Sigma-Aldrich) via the tail vein. Two hours post-injection, animals were euthanized, and transcardial perfusion with ice-cold PBS was performed. Brains were then harvested, weighed, and homogenized in N, N-dimethylformamide (Sigma-Aldrich). The homogenates were incubated at 60°C for 72 hours, followed by centrifugation at 16,000 rpm for 30 minutes at 4°C. The supernatants were collected, and EB concentration was measured by spectrophotometry at 620 nm using a Molecular Devices plate reader. Results were normalized to tissue weight (μg/g).

Neurobehavioral Assessment

Neurological function was assessed using the modified neurological severity score (mNSS) and Rotarod test at 7 days post-TBI by two individuals blinded to the group identity of each mouse, as described previously. The mNSS consists of motor (muscular state and abnormal action), sensory, reflex, and balance tests. A higher mNSS score represents more severe neurological deficits (normal score = 0; maximal score = 18). As described previously, the limb motor coordination and balance of mice were assessed using an accelerating Rota-rod apparatus (RWD Life Science, Shenzhen, China). Before induction of TBI, mice in each group were trained for three consecutive days. Then, at 7 days post-injury, each mouse was placed on the accelerating automated Rota-rod, which accelerated from 4 to 40 rpm/min within 5 minutes. The latency to fall for each mouse was recorded. Each mouse was tested three times with the same speed each day, with an interval of 30 minutes between trials. The average latency to fall was used for analysis.

The Morris water maze was carried out to evaluate spatial learning and memory function of mice at 15 - 21 days post-TBI. The maze was set in a dim environment and consisted of a circular metallic pool (120 cm diameter), filled to a depth of 50 cm with dyed white water using opaque nontoxic paint. The pool was divided into 4 quadrants, with a cylindrical platform (10 cm diameter) placed in the center of one quadrant. The platform was submerged 1 cm below the surface of the water for the mice to search for it. The experiment required continuous training for 7 consecutive days, with the training period lasting from 15 to 20 days and the 21st day serving as the testing period. During the learning phase of the test, each mouse was tested 4 times per day in different quadrants, followed by a swimming period of up to 90

seconds to escape to the hidden platform. During the testing phase, the escape platform was removed, and each mouse was placed into the pool diagonally opposite the platform quadrant and observed for 90 seconds. A video tracking system (EthoVision XT 13, Noldus Information Technology, Wageningen, the Netherlands) was used to record and analyze the swimming traces, escape latency (the time spent finding the platform), platform crossing times, and the number of crossings. Swim speed was also recorded to assess motor skills.

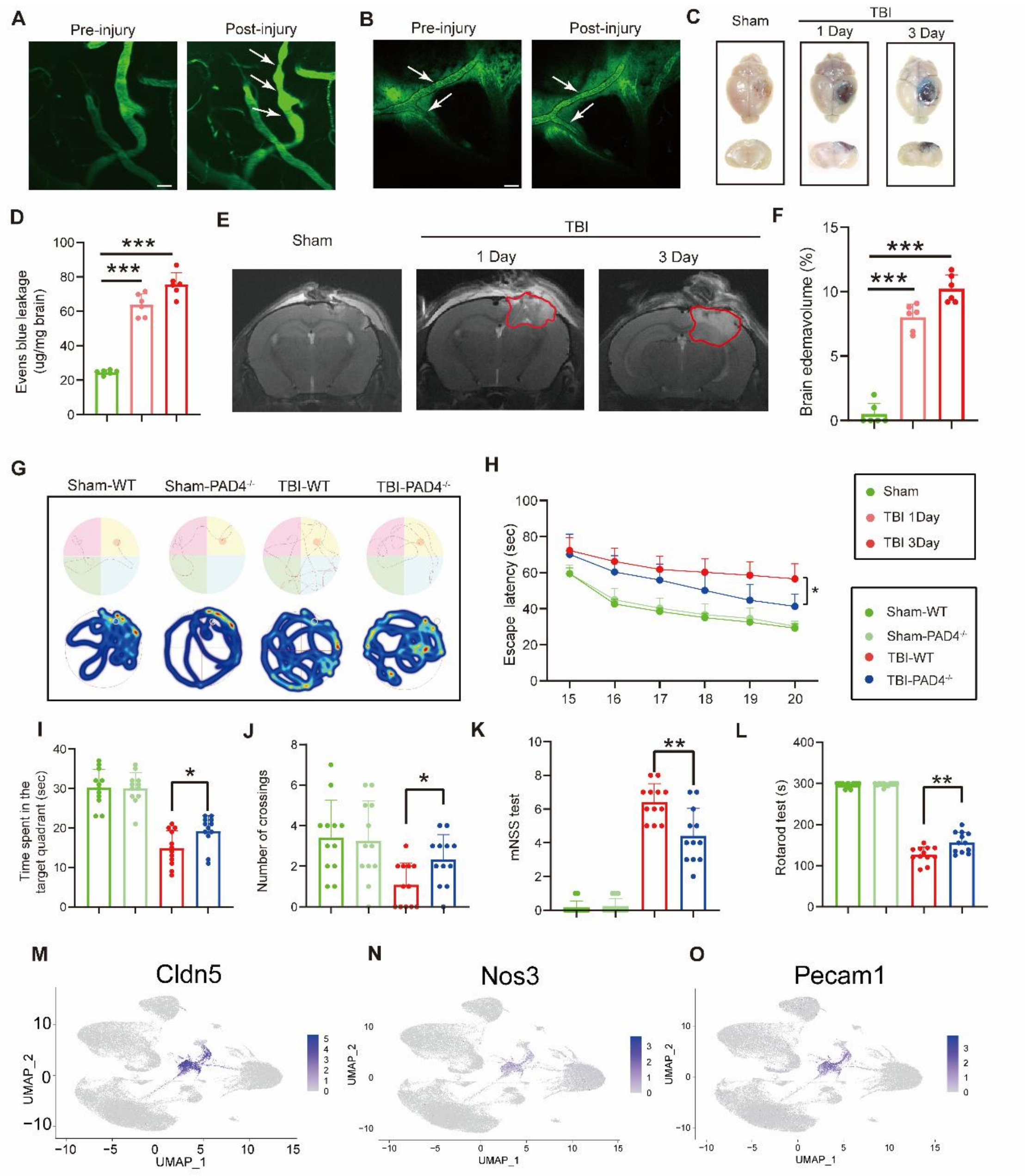

Fig. S1 (A and B) Representative images of the same cortical blood vessel before TBI and on day 3 post-TBI,

showing morphological changes and a reduction in vessel diameter (indicated by white arrows). Vessels labeled with intravenous FITC-dextran (2000 kDa). Scale bar = 100 μm. **(C and D)** Representative horizontal images of mouse brains from different groups after EB injection, along with quantitative analysis of EB leakage intensity. **(E and F)** Representative MRI scans of different groups of mice on day 3 post-TBI, along with quantitative analysis of brain edema volume. **(G)** Representative swim paths of the Morris water maze during the learning and memory phases of the test. **(H)** Spatial learning was assessed by the escape latency of the Morris water maze on days 15–20 after TBI. (n = 6/group). **(I)** The time spent in the target quadrant and **(J)** numbers of the crossing of the target quadrant at 21 d after TBI. (n = 6/group). **(K, L)** Rotarod test and mNSS test on days 7 post-TBI. (n = 12/group). (**M-O**) Expression of endothelial cell-specific markers (Cldn5, Nos3, Pecam1) in the UMAP plot. Data are presented as mean ± SD and analyzed using one-way ANOVA and repeated measures ANOVA with Bonferroni's multiple comparison test. *p < 0.05, **p < 0.01, ***p < 0.001.

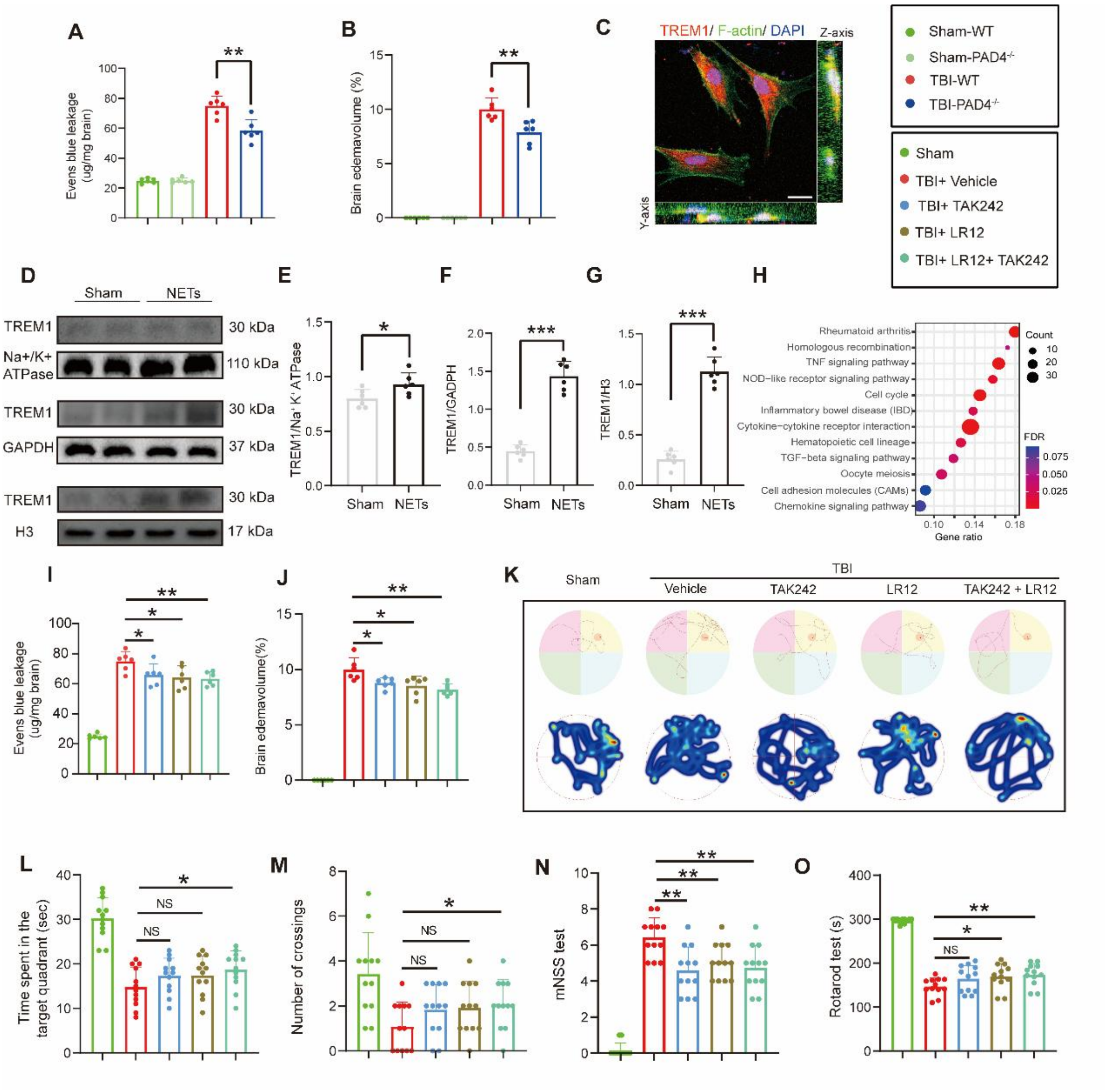

Fig. S2 (A) Mouse brains from different groups on day 3 post-TBI after EB injection, accompanied by quantitative analysis of EB leakage intensity. **(B)** MRI quantitative analysis of brain edema volume in different groups of mice

on day 3 post-TBI. **(C)** Confocal microscopy images showing representative double immunofluorescence staining for TREM1 (red) and F-actin (green). Nuclei were stained with DAPI (blue). bar = 20 μm. **(D and G)** The protein expression levels of TREM1 in endothelial cell membrane, cytoplasmic, and nuclear samples under different treatments were evaluated by Western blot analysis and density quantification. Na^{+} K^{+} ATPase, GAPDH and H3 were used as loading controls, respectively. (n = 6/group). **(H)** KEGG enrichment of differentially expressed genes in NETs+TAK242 vs NETs-treated HUVECs **(I)** Mouse brains from different groups on day 3 post-TBI after EB injection, accompanied by quantitative analysis of EB leakage intensity. **(J)** MRI quantitative analysis of brain edema volume in different groups of mice on day 3 post-TBI. **(K)** Spatial memory was assessed by the escape latency, **(L)** The time spent in the target quadrant and **(M)** numbers of the crossing of the target quadrant at 21 d after TBI (n = 12/group). **(N, O)** Rotarod test and mNSS test on days 7 post-TBI. Data are presented as mean ± SD and analyzed using one-way ANOVA or repeated measures ANOVA with Bonferroni's multiple comparison test. *$p < 0.05$, **$p < 0.01$, ***$p < 0.001$.
